# Very-High Color Rendering Index Hybrid White Organic Light-Emitting Diodes with Double Emitting Nanolayers

**DOI:** 10.1007/s40820-014-0006-4

**Published:** 2014-09-23

**Authors:** Baiquan Liu, Miao Xu, Lei Wang, Hong Tao, Yueju Su, Dongyu Gao, Linfeng Lan, Jianhua Zou, Junbiao Peng

**Affiliations:** 1 Institute of Polymer Optoelectronic Materials and Devices, State Key Laboratory of Luminescent Materials and Devices, South China University of Technology, Guangzhou, 510640 People’s Republic of China; 2 New Vision Opto-Electronic Technology Co., Ltd, Guangzhou, 510530 People’s Republic of China

**Keywords:** White light, Hybrid, Color rendering index, Organic light-emitting diodes, Double emitting nanolayers

## Abstract

A very-high color rendering index white organic light-emitting diode (WOLED) based on a simple structure was successfully fabricated. The optimized device exhibits a maximum total efficiency of 13.1 and 5.4 lm/W at 1,000 cd/m^2^. A peak color rendering index of 90 and a relatively stable color during a wide range of luminance were obtained. In addition, it was demonstrated that the 4,4′,4″-tri(9-carbazoyl) triphenylamine host influenced strongly the performance of this WOLED. These results may be beneficial to the design of both material and device architecture for high-performance WOLED.

## Introduction

White organic light-emitting diodes (WOLEDs) are considered to be one of the most stormily developing technologies nowadays owing to their potential applications in the display markets and next-generation lighting sources [1]. In terms of the practical use, whereas the color rendering index (CRI) is not a critical parameter for OLEDs in full-color displays, it must be considered in addition to Commission International de l′Eclairage (CIE) when designing WOLEDs for lighting since the CRI gives an indication of how well the light source will render colors of objects it illuminates [2].

For a source to be human eye-friendly, a WOLED with a CRI above 80 is required [1]. Since D′Andrade et al. took the first step to demonstrate that the CRI of WOLEDs can be greatly enhanced from 50 to 83 [3], a great deal of attention has been attracted to the improvement of CRI, which is expected to meet the commercial requirements. For instance, Sun et al. developed a hybrid WOLED which combines fluorescent blue emitters with phosphorescent green–red emitters, achieving a CRI of 85 [4]. Park et al. synthesized a yellowish-green dopant to fabricate a three-color phosphorescent WOLED with a CRI of 86.8 [5]. In recent years, in order to satisfy the demand of high-quality lighting systems, such as surgery, photography, and exhibition of museums, WOLEDs with very-high CRI (≥90) are extremely required [6].

As a matter of fact, a few efforts have been explored to the pursuit of very-high CRI WOLEDs. For example, Wang et al. designed a WOLED using exciplex emission from mixed acceptors, achieving a CRI of 90.4 at 11 V (a maximum brightness of 425 cd/m^2^) [7]. Chang et al. constructed a phosphorescent WOLED using a four-emitting-layer and double-confining-layer structure, achieving a CRI of 94 and a forward-viewing power efficiency (PE) of 3.8 lm/W at 1,000 cd/m^2^ [8]. Chen et al. inserted a spacer between the fluorescent emitter and the phosphorescent emitters to realize a hybrid WOLED, achieving a CRI of 91.2 (no efficiency data are reported) [9]. Hao et al. fabricated a WOLED combining emission from excitons and interface-formed electroplex, achieving a CRI of 90.2 at 8 V (a maximum efficiency of 1.39 cd/A) [10]. Jou et al. demonstrated an efficient WOLED using five emitters, achieving a CRI of 96 with 5.2 lm/W at 1,000 cd/m^2^ [6]. Li et al. proposed a WOLED using a four-emitting-layer architecture, achieving a CRI of 97 with 8.19 lm/W at 1,000 cd/m^2^ [11].

Based on these facts [7–11], it can be concluded that various approaches can be used to develop very-high CRI WOLEDs. However, it is easily noted that these very-high CRI devices show either rather poor luminance/efficiency [7, 10] or somewhat complicated structures [6, 8, 9, 11], which limit their further applications. Therefore, to greatly lower the cost and vastly simplify fabrication processes, very-high CRI WOLEDs with simple structures are urgently needed to meet the future commercial demand. Moreover, negligible report has been documented to illustrate the role of the host in the performance of very-high CRI WOLEDs.

In this paper, we developed a very-high CRI WOLED with a hybrid structure which comprises with only two emitting layers. The resulting device exhibits a maximum total PE of 13.1 and 5.4 lm/W at 1,000 cd/m^2^. A peak CRI of 90 is obtained, which represents the highest value in hybrid WOLEDs based on the dual-emitting-layer structure, to the best of our knowledge. Besides, a relatively stable color can be obtained during a large range of luminance (1,000–12,000 cd/m^2^). Moreover, it is demonstrated that the 4,4′,4″-tri(9-carbazoyl) triphenylamine (TCTA) host plays a critical role in realizing such high performance. Such presented results demonstrate that efficient very-high CRI WOLEDs can be effectively realized by using simple structures.

## Experimental

**Fig. 1:**
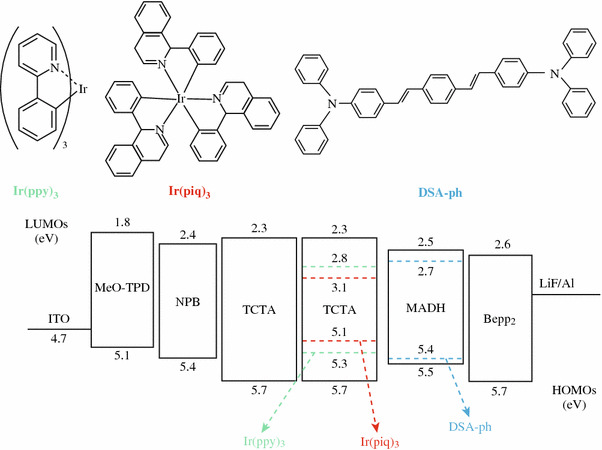
*Top* the chemical structure of emissive dopants. *Bottom* proposed energy-level diagram of the WOLED, showing the highest occupied and lowest unoccupied molecular orbital energies relative to the vacuum level

As vividly depicted in Fig. 1, the configuration of the studied hybrid WOLED (W1) is ITO/MeO-TPD: F4-TCNQ (100 nm, 4 %)/NPB (15 nm)/TCTA (5 nm)/TCTA: Ir (ppy)_3_ : Ir(piq)_3_ (25 nm, 1:9:0.8 %)/MADN: DSA-ph (20 nm, 1 %)/Bepp_2_ (250 nm)/LiF (1 nm)/Al (200 nm), where ITO is indium tin oxide, F4-TCNQ is tetrafluoro-tetracyanoqino dimethane, was doped into *N*, *N*, *N′*, *N′*-tetrakis(4-methoxyphenyl)-benzidine (MeO-TPD, as a hole injection layer), NPB is *N*, *N*′-di(naphthalene-1-yl)-*N*, *N*′-diphenyl-benzidine (as a hole transport layer), TCTA is an exciton barrier layer and a host of phosphorescent emitters, Ir (ppy)_3_ is tris(2-phenylpyridinne)iridium(III) (as a green emitter), Ir(piq)_3_ is tris(1-phenylisoquinolinolato-*C*^2^,*N*) iridium(III) (as a red emitter), fluorescent material p-bis(p-*N*, *N*-di-phenyl-aminostyryl) benzene (DSA-ph) as a blue guest, was doped into 2-methyl-9,10-di(2-naphthyl)anthracene (MADN), Bepp_2_ is bis[2-(2-hydroxyphenyl)-pyridine] beryllium (as an electron transport layer), LiF is an electron injection layer, and Al is a cathode. All materials used were commercially bought. The detailed fabrication and measurement of devices followed well-established processes and as reported elsewhere [12].

## Results and Discussion

**Fig. 2:**
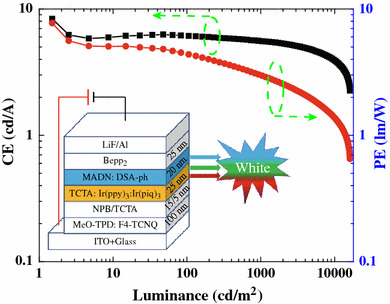
Power and current efficiencies as a function of luminance for the device. *Inset* the structure of the device

The current efficiency (CE) as well as PE of the device W1 in dependence of the luminance are clearly shown in Fig. 2. The maximum forward-viewing CE and PE of the device are 8.4 cd/A and 7.7 lm/W at a luminance of 1.5 cd/m^2^, respectively. At a luminance of 1,000 cd/m^2^, the CE and WPE are 5.6 cd/A and 3.0 lm/W, respectively. Since illumination sources are typically characterized by their total emitted power [4], the maximum total efficiency of the device is 13.1 lm/W, which is comparable to that of incandescent light bulbs (12–17 lm/W). At 1,000 cd/m^2^, the total efficiency is 5.4 lm/W. Apparently, the efficiency of the device is higher than the previous hybrid WOLEDs [13, 14]. Moreover, it is important to note that much higher efficiency can be expected if we replaced the electron transport layer with an n-doped layer to form a p-i-n architecture and utilized out-coupling methods to increase the light extraction.

**Fig. 3:**
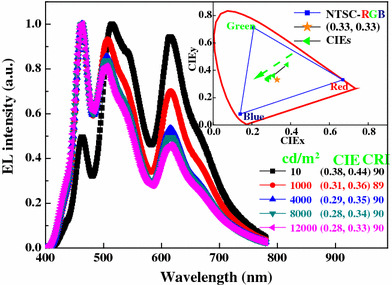
Normalized EL spectra of W1 at various luminances. *Inset* the labeled spots represent CIE coordinates at different luminances

Spectral stability upon change of luminance was studied; a blue emission peak at 462 nm, a green emission at 514 nm, and a red emission at 615 nm are obviously displayed in Fig. 3, covering all wavelengths from 380 to 780 nm. When the luminance increases from 10 to 12,000 cd/m^2^, the CIE coordinates of the device experience a change from (0.38, 0.44) to (0.28, 0.33), indicating that the recombination zone is shifted to the blue emissive region as the luminance/driving voltage increases. This blue-shifted phenomenon can be explained as follows. From Fig. 1, it is seen that the highest occupied molecular orbital (HOMO) of TCTA, Ir(ppy)_3_ and Ir(piq)_3_ are 5.7 eV [15], 5.3 eV [16], and 5.1 eV [17], respectively, implying that a fraction of holes injected from TCTA can be effectively trapped by these two dopants [15]. Hence, at a low luminance, the red and green emission are stronger than blue emission. As the luminance/driving voltage increases, the holes trapped by the phosphorescent dopants should be saturated and more holes can transport to the blue region, resulting in the intensity enhancement of blue emission [18].

More remarkably, a very-high CRI of 90 is obtained at both a low luminance (10 cd/m^2^) and high luminances (4,000–12,000 cd/m^2^). The device exhibits Duvs of 0.0283, 0.0198, 0.0235, 0.0245, and 0.0238 at 10, 1,000, 4,000, 8,000, and 12,000 cd/m^2^, respectively. In addition, the combination of high CRI and the special CRI R9, which quantifies the color reproduction of saturated red and should be positive, is very interesting for commercial applications [19]. Our device shows R9 s of 81, 69, 84, 91, and 92 at 10, 1,000, 4,000, 8,000, and 12,000 cd/m^2^, respectively. Besides, similar to other WOLEDs which show relatively large color-shift at low luminances and relatively slight color-shift at high luminances [12, 20–22], our device exhibits only a little CIE coordinates variation of Δ(*x*, *y*) ≤ (0.03, 0.03) during a wide range of luminance (1,000–12,000 cd/m^2^), revealing that the device exhibits a relatively stable color [20].

It is clearly noted that previous reports are usually focused on the effect of dopants in the very-CRI WOLEDs [6, 8, 9, 11], negligible attention is paid to the influence of hosts. Herein, to explore the working mechanism of the simple device and to illustrate the significance of the TCTA host guaranteeing the high performance, we used a well-known bipolar material 4,4-*N*, *N*-dicarbazolebiphenyl (CBP) to replace TCTA as the host. The configuration of CBP-based device (W2) is ITO/MeO-TPD: F4-TCNQ (100 nm, 4 %)/NPB (15 nm)/TCTA (5 nm)/CBP: Ir (ppy)_3_ : Ir(piq)_3_ (25 nm, 1:9:0.8 %)/MADN: DSA-ph (20 nm, 1 %)/Bepp_2_ (250 nm)/LiF (1 nm)/Al (200 nm).

**Fig. 4:**
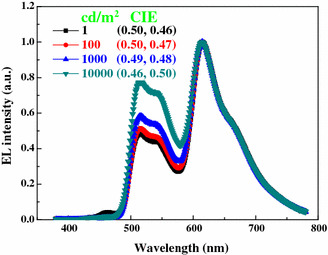
Normalized EL spectra of W2 at different luminances

Figure 4 shows the spectra of W2 as the luminance increase from 1 to 10,000 cd/m^2^. It is obviously noted that only negligible blue emission can be observed in the whole luminances, and hence no white light can be generated. Although bipolar hosts are usually considered to be preferred compared with unipolar hosts [23], our results contradict this previous opinion. This phenomenon may be understood as follows. Since the HOMO of CBP is 6.1 eV [16], a large energy barrier of 0.4 eV exists between the exciton layer (TCTA) and the CBP host, indicating that holes may be prevented to hopping from TCTA to CBP due to the fact that the current flow is limited by the injection of carriers when the injection energy barrier is higher than 0.3–0.4 eV [24, 25]. On the other hand, a part of holes injected to the phosphorescent layer are effectively trapped by the Ir(ppy)_3_ and Ir(piq)_3_ because the HOMO of these two guests is much higher than that of the CBP host [15]. Consequently, only little holes can be transported to the blue region due to the above combined effects, leading to no white color being observed. However, in the case of device W1, no energy barrier located between the exciton layer (TCTA) and the TCTA host, implying that holes are easily transported to the phosphorescent layer. Besides, TCTA is a typical hole-type material [15, 20], indicating that a large number of holes can be transported along the TCTA molecular to reach the blue emitting layer though some holes are trapped by the dopants, guaranteeing the blue emission. Therefore, despite the TCTA is a unipolar host, it plays a key role in the performance of the very-high CRI WOLED.

On the other hand, it can be easily inferred that a narrow recombination zone of device W1 is formed at the phosphorescent emitting layer/flourescent emitting layer interface due to the existed energy barriers [26] together with the fact that TCTA is typical hole-type materials [15, 20] and MADN preferentially transports electrons [27]. The narrow recombination zone can cause intensified triplet-triplet and triplet-polaron interactions at high luminances [28], which not only reduces the efficiency of phosphor-based devices (W1), but also results in an efficiency roll-off of device W1 with increasing luminance [29, 30].

Finally, it should be pointed out that spacers are usually needed to be inserted between the fluorescent- and phosphorescent-emitting layers to realize hybrid WOLEDs, otherwise, no white light can be generated [4, 9, 15, 30, 31]. Herein, we, for the first time, report a very-high CRI hybrid WOLED without a spacer to separate the fluorescent and phosphorescent emitting layers. We hope more efficient very-high CRI devices can be developed in this simple and new way.

## Conclusion

In summary, we have successfully demonstrated a very-high CRI WOLED based on double emissive nanolayers. The resulting device exhibits a maximum total efficiency of 13.1 lm/W and an efficiency of 5.4 lm/W at 1,000 cd/m^2^. A peak CRI of this simple device is as high as 90 and a relatively stable color during a wide range of luminance can be obtained. In addition, it is demonstrated that the TCTA host plays a key role in the performance of this WOLED. Undoubtedly, such achieved results would provide an instructive guide for the rational design of ultra high-performance OLEDs, which will be very advantageous for the commercialization of solid-state lighting in the near future.
